# Older men and older women remand prisoners: mental illness, physical illness, offending patterns and needs

**DOI:** 10.1017/S1041610214002348

**Published:** 2014-11-27

**Authors:** Mary Davoren, Mary Fitzpatrick, Fintan Caddow, Martin Caddow, Conor O’Neill, Helen O’Neill, Harry G. Kennedy

**Affiliations:** 1 National Forensic Mental Health Service, Central Mental Hospital, Dundrum, Dublin, Ireland; 2 Trinity College Dublin, Dublin, Ireland

**Keywords:** prisons, prisoners, elderly, men, women

## Abstract

**Background:**

Older prisoners are the fastest growing group of prisoners in most countries. They have high rates of physical and psychiatric co-morbidity, compared to community dwelling older persons and also compared with other prisoner groups. Very high rates of mental illness have been found in remand (pre-trial) prisoners when compared with other prisoner groups; however to date there have been no studies examining older male and female remand prisoners.

**Methods:**

A retrospective chart review was conducted of all remands, to a male and a female prison, over a six and half-year period. Demographic data were collected pertaining to psychiatric and medical diagnoses and seriousness of offending.

**Results:**

We found rising numbers of older prisoners amongst male remand prisoners. Older remand prisoners had very high rates of affective disorder and alcohol misuse. They had rates of psychotic illnesses and deliberate self-harm comparable to younger remand prisoners. High rates of vulnerability were found among older prisoners and older prisoners had a greater need for general medical and psychiatric services than younger prisoners. We also found comparable offending patterns with younger prisoners and high rates of sexual offending among the older male prisoner group.

**Conclusions:**

Given the ageing population of many countries it is likely the numbers of older prisoners will continue to grow and given their high levels of both physical and psychiatric illness this will have implications for future service delivery.

## Introduction

### Background

Older prisoners have been classified as “special needs prisoners” (United Nations, 2009). They are a growing group within the prison setting and due to the ageing population in almost all countries of the world, it is likely that their numbers will continue to grow (United Nations Department of Economic and Social Affairs, Population division, 2009; 2013). A 2012 report by Human Rights Watch “Old behind bars,” stated that between 2007 and 2010 in the United States the number of sentenced prisoners aged 65 years or older grew 94 times faster than the total sentenced prisoner population (Human Rights Watch, 2012). In 1995 prisoners over 55 years of age made up 3% of the US prison population, but by 2010 this had risen to 8% of the prison population (Human Rights Watch, 2012). A recent thematic review by the Prison Reform Trust in the UK “No problems old and quiet” found that the over 60s were the fastest growing group in prisons in England and Wales (H.M. Chief Inspector of Prisons, 2004). Between 1990 and 2000 the sentenced prisoner population in England and Wales grew by 51% while the sentenced prisoner population aged 60 years and over grew by 216% (H.M. Chief Inspector of Prisons, 2004). The number of male prisoners in England and Wales over the age of 60 increased three-fold between 1996 and 2008 (H.M. Chief Inspector of Prisons, 2008). Similar trends have been found in Australia (Australian Bureau of Statistics, 2010) and Canada (Public Works and Government Services Canada, 2012).

High rates of mental illness are found in prisons (Fazel *et al.*, 2002; Brugha *et al.*, 2005). A systematic review of 23,000 prisoners in 12 countries found that 3.7% of male prisoners were suffering from psychotic illnesses and that prisoners were several times more likely to suffer from psychosis or major depression than persons in the community (Fazel *et al.*, 2002). Brugha *et al* found that the weighted prevalence of psychosis among prisoners was ten times higher than in the community, with a prevalence rate of psychosis of 4.5 per thousand in a household survey compared to 52 per thousand in the prison survey (Brugha *et al.*, 2005). Remand (pre-trial) prisoners have especially high rates of mental illness when compared with the general population and even when compared with other prisoner groups (Maden *et al.*, 1995; Brooke *et al.*, 1996). High rates of mental disorder and psychosis have also been found in female remand prisoners in a UK study (Parsons *et al.*, 2001). Similarly, high rates of mental illness have been found among remand prisoners in the Republic of Ireland. Curtin *et al* found a six month prevalence rate of psychosis of 3.8% at the point of committal to a male remand prison in Dublin (Curtin *et al.*, 2009).

There is much variation in the age thresholds used to define older prisoners. Older prisoners have significantly higher rates of physical and psychiatric co-morbidities compared to younger prisoners and also compared to elders in the community (Baillargeon *et al.*, 2000; Fazel *et al.*, 2001a). Taylor *et al.* found that older male prisoners were significantly more likely to suffer from physical and psychiatric illness than younger male prisoners (Taylor and Parrott, 1988). The rates of hypertension, diabetes and arthritis in prisoners over the age of 50 years were double the rates of those illnesses found among younger prisoners (Baillargeon *et al.*, 2000) while 85% of older prisoners have at least one chronic illness, the most common being psychiatric or respiratory (Fazel *et al.*, 2001a). Colsher *et al.* found higher rates of chronic physical illnesses for example hypertension, diabetes and emphysema and higher rates of having previously suffered a myocardial infarct or stroke among prisoners over the age of 60 years when compared with prisoners aged between 50 and 59 years (Colsher *et al.*, 1992). Her Majesty's Inspector of Prisons (UK) in 2008 reported that over half of elderly prisoners suffer from a mental illness, the most common being depression (H.M. Inspectorate of Prisons 2008). Fazel *et al.* found a 30% rate of depression among older male prisoners in the UK, which is higher than the rate among younger male prisoners and older persons in the community (Fazel *et al.*, 2001b). Older prisoners have a high rate of suicide after release from prison (Pratt *et al.*, 2006). It has been suggested that prisoners age earlier than the general population and for this reason a lower threshold should be set when considering older prisoners (Uzoaba, 1998; United Nations, 2009; Kakoulis *et al.*, 2010).

Offending patterns differ between older and younger prisoners. A UK in 2008 found that older prisoners had lower rates of non-violent offences but similar rates of violent offences when compared with younger prisoners (Home Office U.K., 2008). In several jurisdictions high rates of sexual offending have been found among the older male prisoner group when compared to younger prisoners; however sexual offences are rare among older female prisoners. In Canada, older prisoners were most often sentenced for sexual offences (Uzoaba, 1998). Fazel *et al.* reviewed male prisoners over the age of 60 years in a UK sample and found that 48.8% were imprisoned for a sexual offence (Fazel *et al.*, 2001b).

### Rationale

There are a number of legal drivers which apply to older persons in prison settings. The United Nations principles for older people state that “Older persons should be able to live in dignity and security, be free of exploitation and physical or mental abuse and be treated fairly regardless of age, gender and racial or ethnic background” and “Older persons should have access to healthcare to help them maintain or regain the optimal level of physical, mental and emotional wellbeing and to prevent or delay the onset of illness” (U.N., 1991). The European Prison Rules 2006, which are based on the United Nations standard minimum rules for the treatment of prisoners, state that “Persons who are suffering from mental illness and whose state of mental health is incompatible with detention in a prison should be detained in an establishment specially designed for the purpose” (U.N., 1955; Council of Europe, 2006). A lack of adequate health care in prisons may contravene Article 3 of the European Convention on Human Rights (ECHR) the right to freedom from torture, degrading or inhuman treatment or punishment (European Court of Human Rights, 2010). The Committee for Prevention of Torture of the Council of Europe sets out a standard of equivalence of healthcare, that prisoners must have access to the same standard of healthcare as is available in the community setting (Committee for prevention of torture and inhuman or degrading punishment, 2006).

### Objectives

Because of the high rates of mental illness found in remand prisons and the high rates of both physical and mental illness found internationally in older prisoners, together with the paucity of literature on this group in a pre-trial prison setting, we decided to review the older prisoners on remand in male and female prisons. We hypothesized that we would find growing numbers of elderly prisoners on remand and that those prisoners would have high rates of physical and psychiatric illness compared to younger remand prisoners. It was intended that the information obtained would highlight the needs of older prisoners and may influence the delivery of prison mental health services to ensure optimum care for this group.

## Methods

### Study design

The design of this study is descriptive and retrospective in nature. Every person aged 60 or over was selected with a comparison group selected as the next person admitted to the same prison, to ensure gender balance between older prisoners and comparison groups. We first conducted a retrospective chart review of all those remanded from freedom to two Irish remand prisons, one male and one female. We examined all remands from freedom to these prisons over a six and a half year period from 1^st^ January 2006 until 30^th^ June 2012. Demographic data and data pertaining to the medical and psychiatric characteristics of the prisoners were gathered. We also identified the charge or offence for which the prisoner was remanded in custody.

### Setting

This study was set in two Irish remand prisons. Cloverhill Prison is a medium security closed prison, which accepts male remands from the Dublin and Leinster areas, about 62% of the national population. The Dochas Centre female prison, on the Mountjoy Campus, accepts female remand and sentenced prisoners.

The study was approved by the local research, audit, ethics and effectiveness committee in the National Forensic Mental Health Service, Central Mental Hospital Dundrum Dublin and also by the prisoner research ethics committee of the Irish Prison Service.

### Participants

An age threshold of 60 was adopted in keeping with practice in other published surveys of older prisoners. Prisoners are known to adopt lifestyles that are hazardous to physical and mental health, including use of tabacco, alcohol and street drugs. They may therefore be liable to premature aging (Fazel *et al.*, 2001b; Curtin *et al.*, 2009)

### Variables

Demographic data, data pertaining to medical and psychiatric diagnosis, medications prescribed, placement within the prison setting, length of remand and the offence the prisoner was charged with were gathered from prison records.

#### Seriousness of offences

The DUNDRUM toolkit is a structured professional judgement instrument comprising five scales (Kennedy *at al*., 2010). DUNDRUM-1 triage security scale and DUNDRUM-2 triage urgency scale are designed to assist clinicians in making decisions when allocating a patient to be admitted to hospital at a particular level of therapeutic security (Kennedy *et al.*, 2010). It has previously been shown that the DUNDRUM-1 triage security scale predicts clinical decisions concerning need for therapeutic security among patients on a forensic psychiatry hospital waiting list and the DUNDRUM-2 triage urgency scale predicts clinical decisions regarding the urgency of that need (Flynn *et al.*, 2011).

The DUNDRUM-1 triage security scale consists of 11 items, each of which is rated “0” to “4” where “0” represents no need for hospital admission and “4” represents a need for high security. Each score is tethered to a series of definitions, to ensure consistency and reliability when making ratings. DUNDRUM-1 Triage security item one (D-1 TS1), rates seriousness of recent violence. We noted the offence leading to remand for each prisoner and rated the offences “0” to “4,” using the DUNDRUM-1 Triage security item 1, (Table 1), to compare seriousness of offending between the older prisoners and the younger control groups. When a prisoner was remanded on multiple charges, we rated the most serious charge.

**Table 1.** Dundrum-1 triage security scale

score	definition
**4**	Homicide OR
	Stabbing penetrates body cavity OR
	Fractures skull OR
	Strangulation OR
	Serial serious (e.g. penetrative, indictable) sexual assaults OR
	Kidnap OR
	Torture OR
	Poisoning.
**3**	Use of weapons to injure OR
	Arson endangering life OR
	Assaults causing concussion or fractures to long bones OR
	Stalking with threats to kill OR
	Single serious sexual assault, (indictable).
**2**	Repetitive assaults causing injury such as bruising, that cannot be prevented by two-to-one nursing in open conditions OR
	Less serious sexual assaults, (summary offence).
**1**	Minimal degree of violence and minimal threat to life.
**0**	No previous violence.

Coding: Triage security Item 1 – seriousness of violence: Each individual is rated “0” to “4” on seriousness of previous violence.

### Bias

No data were missing.

### Study size

A total of 22,608 remands to prison from freedom were reviewed, which included a total of 20,084 (89%) males remanded to Cloverhill Prison and 2,524 (11%) females remanded to Dochas Centre over a six and a half year period from 1^st^ January 2006 to the 1^st^ July 2012.

### Statistical methods

All data was entered into SPSS version 21 (IBM Corp., 2012). χ^2^ statistical tests were used to compare the two groups of prisoners.

## Results

### Participants

Of the 22,608 remanded prisoners, 213 were over the age of 60 years at the time of remand. Of the 213 older remands, the majority were males 157 (74%) compared with 56 (26%) females. Females were overrepresented in the older prisoner group, which was not unexpected given that the global population of older persons is predominantly female, as older women live longer than older men (3). Overall 0.8% of male remands were over the age of 60 years, however 2% of all female remands were over the age of 60 years. Similarly, 11% of the total remanded prisoners were female, however 27% of older remands were female (Table 2). The number of older male prisoners committed per annum doubled between 2006 and 2011, while the number of older females remained relatively stable. (Table 3).

**Table 2.** Total numbers of prisoners on remand January 2006–June 2012

	male	female	total
Younger remand prisoners	19,927	2,468	22,395
Older remand prisoners	157	56	213
Total numbers of remand	20,084	2,524	22,608
prisoners

**Table 3.** Numbers of male and female older prisoners remanded each year 2006–2011 and 1st January until 30th June 2012

	2006	2007	2008	2009	2010	2011	*2012 (Figures for 6-month period)*	Total
Older males	17	17	18	25	27	38	*15*	157
Older females	10	9	10	9	6	10	*2*	56
Total older prisoners	17	16	28	34	33	48	*17*	213

### Main results

Among the older prisoner group, the mean age at committal was 64.5 years, median 63.1, mode 64.6 years and standard deviation 4.2 years. Among the 213 older prisoners, the majority (190 prisoners) were aged between 60 and 69 years, 21 older prisoners were aged between 70 and 79 years and the two oldest prisoners were in their eighties at the time of committal. For the younger prisoner group, mean age at committal was 31.9 years, median 31.0 years, mode 21.0 years and standard deviation 9.1 years.

Older prisoners had similar rates of psychosis to younger prisoners. 4 (2%) of the older remands had a diagnosis of a psychotic disorder compared to 9 (4%) of the younger group (X^2^ = 2.419, df = 2, *p* = 0.298). All 13 with a history of psychotic illness were male remand prisoners. Affective disorders were significantly more common among older prisoners compared with the younger prisoner control group. 80 (38%) of older remands had a history of affective disorder compared to 36 (17%) of the younger control group (X^2^ = 23.356, df = 2, *p* <0.001). Female remands had more affective disorder (18 (32%)) than younger female controls (9 (16%)) though the difference did not reach statistical significance (X^2^ = 3.954, df = 2, *p* = 0.139). For older male remands 62 (40%) had a diagnosis of affective illness, compared with 27 (17%) of younger male controls (X^2^ = 19.810, df = 2, *p* <0.001), (Table 3).

There was no significant difference in the rates of self-harm between the older group and the younger group studied. A documented history of self-harm was found in 25 (12%) older remands and 32 (15%) younger remands (X^2^ = 1.518, df = 2, *p* = 0.468).

Both groups had similar rates of substance misuse however the older group were more likely to misuse alcohol and the younger group more likely to misuse illicit drugs. A history of substance misuse was found in 83 (39%) older prisoners compared to 81 (38%) younger prisoners, (X^2^ = 0.311, df = 2, *p* = 0.856). Older prisoners were significantly more likely to have a documented history of alcohol misuse, 77 (36%) compared with 34 (16%) younger prisoners (X^2^ = 22.889, df = 2, *p* <0.001). Younger prisoners were significantly more likely to have a documented history of illicit drug misuse, 16 (8%) older prisoners, compared with 69 (32%) among the younger control group (X^2^ = 44.876, df = 2, *p* <0.001). This pattern continued when we examined the male prisoner groups but differed among the female prisoner groups. Older male prisoners were significantly more likely to misuse alcohol than younger male prisoners (X^2^ = 31.238, df = 2, *p* <0.001) and older male prisoners were significantly less likely to misuse illicit drugs than younger male prisoners (X^2^ = 36.416, df = 2, *p* <0.001). Among the female prisoner groups there was no significant difference in rates of alcohol misuse between older female prisoners and younger female prisoners (X^2^ = 0.751, df = 2, *p* = 0.687) although older female prisoners were less likely to misuse illicit drugs than younger female prisoners (X^2^ = 9.022, df = 2, *p* = 0.011), (Table 4).

**Table 4.** Physical and psychiatric morbidity among older and younger prisoners

	male prisoners		female prisoners		
	older	younger	older	younger	
	prisoner	control	prisoner	control	total
Psychotic disorder	4 (3%)	9 (6%)	0	0	13
Affective disorder	62 (40%)	27 (17%)	18 (32%)	9 (16%)	116
Substance misuse (any)	72 (46%)	61(39%)	11 (20%)	20 (36%)	164
Alcohol misuse	72 (46%)	27 (17%)	5 (9%)	7 (13%)	111
Illicit drug misuse	10 (6%)	51 (32%)	6 (11%)	18 (32%)	85
Neurological illness	43 (27%)	20 (13%)	6 (11%)	4 (7%)	73
Cardiac illness	28 (18%)	4 (3%)	2 (4%)	1 (2%)	35

We examined whether the prisoners had a documented history of neurological illness for example a history of seizures, epilepsy, head injury with loss of consciousness or Wernicke's encephalopathy. We found high rates of neurological illness in both prisoner groups, particularly so among older prisoners. A neurological illness was found in 49 (23%) older remands compared to 24 (11%) younger remands (X^2^ = 10.333, df = 2, *p* = 0.006). By gender, 43 (27%) older male remands had a diagnosis of neurological illness, compared to 20 (13%) of younger male remands (X^2^ = 10.602, df = 2, *p* = 0.005) but there was no significant difference in history of neurological disorder between older and younger female remands (X^2^ = 0.622, df = 2, *p* = 0.733). (Table 4). We examined whether the prisoners had a documented history of cardiac illness for example a history of angina, ischemic heart disease or congestive cardiac failure. We found that 30 (14%) older remands had a documented diagnosis of a cardiac illness compared to 5 (2.3%) of the younger control group (X^2^ = 19.471, df = 2, *p* <0.001). Again we found that older male remands were significantly more likely to have a documented history of cardiac illness compared with younger male controls (X^2^ = 20.237, df = 2, *p* <0.001) however there was no significant difference between older female remands and younger female remands (X^2^ = 0.567, df = 2, *p* = 0.763). (Table 4). We found that the proportion of older prisoners taking prescribed medication was significantly higher than the younger control prisoner group. Older prisoners were taking prescribed medication in 149 (70%) cases compared with 97 (46%) of the younger control group (X^2^ = 26.014, df = 1, *p* <0.001).

Whether or not a prisoner has a fixed abode is documented on reception to a prison by the committing officer. We examined rates of homelessness among the two prisoner groups. We found that overall rates of homelessness did not differ significantly between the two groups, 61 (28.6%) elderly remands were homeless compared to 62 (29.1%) of the younger control group (X^2^ = 0.011, df = 1, *p* = 0.915). At the committal interview the prisoner officer, together with prison nursing staff also assess the type of accommodation within the prison setting that they consider will best meet that prisoners needs during their committal. We found that 102 (48%) of older remands were deemed to required high support accommodation in the prison on committal, compared with 52 (24%) of the younger control group (X^2^ = 49.187, df = 2, *p* <0.001). We also compared the mean length of stay (in days) in prison high support accommodation during the course of the remand and found that older prisoners had significantly longer lengths of stay in high support accommodation than the younger control prisoner group (ANOVA F = 1.533, df = 34, *p* = 0.032).

We examined prison notes to establish if there was any documented history of victimization within the prison setting for each of the prisoners in the older prisoner and younger prisoner groups, for example having suffered assaults intimidation or threats during the period in custody. We found that 38% of older prisoners had a documented history of such victimization or bullying in the prison setting compared to 12% of younger prisoners (X^2^ = 40.578, df = 2, *p* <0.001).

We graded the seriousness of the offence for which the prisoners were remanded using the DUNDRUM-1 triage security item 1, seriousness of previous violence (Kennedy *et al.*, 2010). Using this scale, each offence is rated from “0” to “4,” with zero being the least serious offences and four being the most serious offences. Each score is tethered to a series of definitions to ensure consistent ratings, as seen in Table 1. When we compared the seriousness of the offences the two prisoner groups were remanded for, we found little difference between the older and younger prisoner groups and many of the older prisoners had been remanded for serious offences (Tables 5 and 6). We then examined whether or not there was a difference in history of sexual offending between the older and the younger prisoner groups. We found that 35 (16%) older prisoners had a history of sexual offending compared to 4 (2%) of the younger control group X^2^ = 34.234, df = 2, *p* <0.001. All those with a history of sexual offending were male prisoners and 89% of those prisoners who had a history of sexual offending were male prisoners over age 60 years.

**Table 5.** Seriousness of offences divided by age group

	seriousness of offences rated using dundrum-1 triage						
	security item 1 (seriousness of violence)						
	d-1	d-1	d-1	d-1	d-1	*offence*	
	score = 0	score = 1	score = 2	score = 3	score = 4	*unknown*	total
Older prisoner	27 (13%)	109 (51%)	23 (11%)	17 (8%)	3 (1%)	*34 (16%)*	213
(% within older)							
Younger prisoner	62 (29%)	92 (43%)	25 (12%)	10 (5%)	2 (1%)	*22 (10%)*	213
(% within younger)							
All prisoners	89 (21%)	201 (47%)	48 (11%)	27 (6%)	5 (1%)	*56 (13%)*	426
(% within all prisoners)

**Table 6.** Seriousness of offences divided by age group and gender

	seriousness of offences rated using DUNDRUM-1 (D-1) triage						
	security item 1 (seriousness of violence)						
	d-1	d-1	d-1	d-1	d-1	*Offence*	
	score = 0	score = 1	score = 2	score = 3	score = 4	*Unknown*	total
Older males	14 (9%)	76 (48%)	21 (13%)	17 (11%)	2 (1%)	*27* (17%)	157
Younger males	42 (27%)	68 (43%)	21 (13%)	8 (5%)	1 (1%)	*17* (11%)	157
Older females	13 (23%)	33 (59%)	2 (4%)	0 (0%)	1 (2%)	*7* (13%)	56
Younger females	20 (36%)	24 (43%)	4 (7%)	2 (4%)	1 (2%)	*5 (9%)*	56

## Discussion

### Key results

This is the first study of older prisoners on remand (pre-trial) that we know of. We found rising numbers of older prisoners year on year in the male remand prison setting. We found very high rates of affective disorder and alcohol misuse among this group. We also found high rates of cardiac and neurological disorders among older prisoners. We found that older remand prisoners had high rates of psychotic illnesses and deliberate self harm, comparable to younger remand prisoners.

We found high rates of vulnerability and victimization among older prisoners and older prisoners had a greater need for general medical and psychiatric services than younger prisoners. The older prisoners had a demonstrated increase in need for dependency, spending a disproportionate amount of time in high support accommodation in the prisons. We believe this supports the use of an age threshold of 60 to identify a group with distinct needs for physical and mental health care.

We also found comparable offending patterns with younger prisoners and high rates of sexual offending among the older prisoner group.

### Limitations

The main limitation of this study is that it is retrospective in nature. Our data were obtained from a retrospective chart review of previously made diagnoses and consequently we believe we may have underestimated the prevalence of illness among older prisoners. This may have been a particular issue in relation to the prevalence of victimization of older prisoners as we were relying on episodes documented by prison staff, which may well have underestimated the prevalence of intimidation among this vulnerable group. While some of the excess of victimization may be due to the numbers of older prisoners charged with sexual offences, it is the absolute level of victimization that matters, not the reasons for it. Older people are also likely to be less physically and mentally resilient in the face of violence and other victimization.

It is possible that there is a tendency amongst prison medical and nursing staff to be more sensitive to mental and physical illness in older prisoners, while under-estimating this in younger prisoners. It is possible also that older prisoners are more willing to seek medical help than younger prisoners, particularly amongst men. However the overall rate of psychosis that we found documented corresponds well with a rigorous epidemiological survey of remand committals in the same prisons (Curtin *et al.*, 2009) so the effects of any bias are likely to be minor. A true prospective survey would be justified however.

Another limitation is the small number of older female prisoners in the group studied, however these small numbers also reflect reality as older women make up very small numbers within the prison populations generally.

### Interpretation

We found high rates of mental illness in the group of older prisoners in two remand prison settings. We think the high rates of morbidity in older prisoners are particularly significant because they are higher than a control group of younger remand prisoners, a group known to have high rates of mental illness in many jurisdictions. This group also required greater use of high support accommodation in prison. Although the absolute numbers identified are as yet small, the increasing trend is notable. While the psychosis rate is lower amongst older prisoners the prevalence of affective disorders, physical illnesses and vulnerability to victimization is much higher, particularly amongst older men. Given the ageing population of most western countries we think that this group will continue to grow and given their high levels of both physical and psychiatric illness we think this will have implications for future service delivery.

### Generalizability and conclusions

Remand prisons typically deal with higher psychiatric morbidity than other prisons (Fazel and Danesh, 2002). We have identified a different pattern amongst older remand prisoners with higher rates of affective illness and alcohol problems, along with higher rates of physical illness and victimization. Prospective studies are now required, and there is a need for a screening protocol specific to the needs of older prisoners. Our findings are similar to the results for sentenced prisoners found in other jurisdictions and it is likely that this will have implications for future service development and delivery. Prisons almost always have special accommodation for vulnerable prisoners e.g. sex offenders. We have identified a new type of vulnerable prisoner and we believe that a new type of vulnerable prisoner unit is now needed, together with improved prison health and mental health services dedicated to the needs older prisoners.

## Conflict of interest

None.

## Description of authors’ roles

MD and HGK designed the study. MD gathered the data. MD and HGK completed the statistical analysis. All contributed to the authorship of the paper.
